# An *N*-Port Universal Multimode Optical Router Supporting Mode-Division Multiplexing

**DOI:** 10.3390/mi12121438

**Published:** 2021-11-25

**Authors:** Yuehong Yang, Ye Su, Bocheng Liu, Junxiong Chai, Li Dai, Xiao Jiang, Yichen Ye, Tingting Song, Yiyuan Xie

**Affiliations:** 1 School of Electronics and Information Engineering, Southwest University, Chongqing 400715, China; yangyh12@foxmail.com (Y.Y.); su895268004@gmail.com (Y.S.); lbc0367@email.swu.edu.cn (B.L.); jxchai@email.swu.edu.cn (J.C.); daili318@foxmail.com (L.D.); jx9450@163.com (X.J.); ycye451@swu.edu.cn (Y.Y.); 2 School of Computer and Information Science, Chongqing Normal University, Chongqing 401331, China; ttsong_53@163.com

**Keywords:** multimode optical router, multimode switching element, mode (de)multiplexer, transmission loss, crosstalk

## Abstract

Optical network-on-chip (ONoC) is based on optical interconnects and optical routers (ORs), which have obvious advantages in bandwidth and power consumption. Transmission capacity is a significant performance in ONoC architecture, which has to be fully considered during the design process. Relying on mode-division multiplexing (MDM) technology, the system capacity of optical interconnection is greatly improved compared to the traditional multiplexing technology. With the explosion in MDM technology, the optical router supporting MDM came into being. In this paper, we design a multimode optical router (MDM-OR) model and analyze its indicators. Above all, we propose a novel multimode switching element and design an *N*-port universal multimode optical router (MDM-OR) model. Secondly, we analyze the insertion loss model of different optical devices and the crosstalk noise model of *N*-port MDM-OR. On this basis, a multimode router structure of a single-mode five-port optical router is proposed. At the same time, we analyze the transmission loss, crosstalk noise, signal-to-noise radio (OSNR), and bit error rate (BER) of different input–output pairs by inputting the 1550 nm TE${}_{0}$, TE${}_{1}$, and TE${}_{2}$ modes to the router.

## 1. Introduction

Optical network-on-chip (ONoC) is a new interconnection technology that replaces electrical connections with optical connections, overcoming the shortcomings of traditional electrical interconnections, and has broad application prospects in high-speed communication networks due to its low latency, ultra-high bandwidth, and distance-independent power consumption [1,2,3,4]. In order to further improve the channel capacity of optical interconnection, many (de)multiplexing technologies have been proposed and studied, such as wavelength-division multiplexing (WDM) [5,6,7,8], code-division multiplexing (CDM) [9,10,11], and time-division multiplexing (TDM) [12,13,14]. Among these multiplexing technologies, the research on WDM technology is the most extensive. However, studies in recent years have shown that WDM technology has been unable to meet the rapidly growing demand for ultra-high bandwidth between inter-chips and on-chips.

From Shannon’s formula theory, it can be seen that the capacity of a single-mode transmission communication system has a limit value. In addition, with the continuous development of multiplexing technologies such as WDM technology, the communication capacity of the communication system is gradually approaching its limit. Since different orthogonal optical modes can be transmitted together in a multimode waveguide, the mode-division multiplexing (MDM) technology can further improve the transmission capability of ONoC. Additionally, compared with WDM, MDM only requires a laser light source of one wavelength and omits accurate wavelength control strategies [15]. Therefore, the mode division multiplexing technology is considered to be a very promising technology, which can provide scalable bandwidth growth for data centers to meet future network requirements. In recent years, many optical devices for mode multiplexing have been studied, such as mode (de)multiplexers [16,17,18,19,20], multimode bendings [21,22], multimode waveguide crossings [22,23,24], multimode switches [25,26,27], and mode filters [28,29].

ONoC is composed of on-chip optical interconnects and routers. On-chip optical router, which is a switching fabric build by microring resonators (MRs) and optical waveguides, is undoubtedly one of the critical components of ONoC. It implements routing and flow control functions and exchanges data packets from the input port to the output port through the optical router unit due to photonic technology. With escalating demands for higher throughput in data centers, compared with traditional optical routers, the optical router support MDM is attractive to parallel chip multiprocessors due to their high bandwidth advantages. However, the multimode optical router (MDM-OR) has not yet formed systematic research results. Therefore, research on MDM-OR is of great significance to the construction of ONoC that supports MDM in the future.

In this paper, based on some mode multiplexed optical devices, the MDM-OR model is proposed. Using the MDM-OR model, we can design different MDM-ORs that support TE${}_{0}$ and arbitrary high-order mode transmission corresponding to different single-mode optical routers. Then, we explain the principle of the MDM-OR model and propose the insertion loss models of different mode multiplexed optical devices. In addition, the crosstalk noise, signal-to-noise radio (OSNR), and bit error rate (BER) of the port pairs of these MDM-ORs are analyzed. Finally, we take the optimized single-mode five-port optical router [29] as an example to show the structure of its corresponding MDM-OR. We input the TE${}_{0}$, TE${}_{1}$, and TE${}_{2}$ modes of 1550 nm to the router, and the transmission loss, the crosstalk noise, the OSNR, and the BER of the port pairs of the MDM-OR are analyzed. The simulation results show that the designed MDM-OR supporting MDM has good performance.

## 2. Materials and Methods

**Figure 1:**
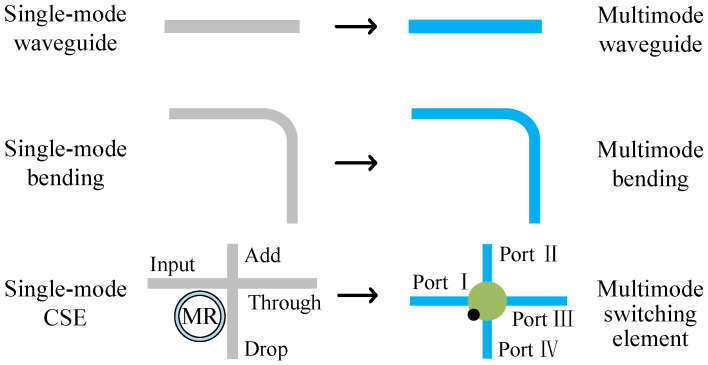
Replacement scheme of component devices of optical transmission unit supporting mode multiplexing.

An optical router unit has the function of transmitting and steering the optical signals; therefore, it is very important to design the universal optical router to realize MDM. In the design of a traditional single-mode router, the optical router consists of single-mode optical waveguides and basic optical switching devices. Similarly, MDM-OR, which allows multimode transmission, is composed of multimode waveguides and basic optical switching devices. These devices include multimode bendings and multimode switching elements. Figure 1 is the general design scheme of component devices of optical transmission unit supporting mode multiplexing. We use multimode waveguide to replace the traditional single-mode waveguide, multimode bending to replace the single-mode bending, and multimode switching element to replace the 1×2 crossing switching element (CSE). These basic optical switching devices based on multiple modes are analyzed in detail below.

**Figure 2:**
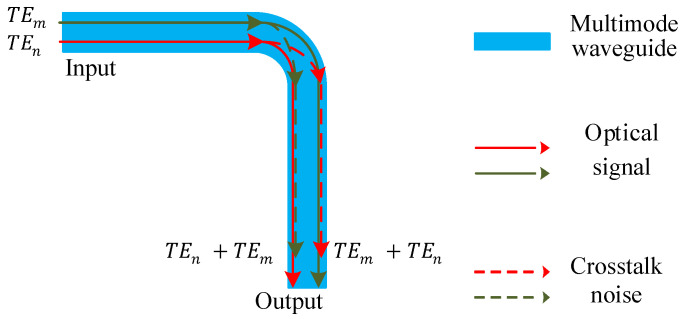
Multimode bending.

Figure 2 shows the multimode bending. The insertion loss is generated when the input signal of multimode bending is TE${}_{0}$, TE${}_{1}$, …, TE${}_{x}$ mode. Unlike the single-mode bending, the multimode bending can transmit TE${}_{0}$, TE${}_{1}$ and other modes simultaneously. When different modes pass through multimode bending, the corresponding loss is also different, which can be calculated by Equation (1). The $P_{in}$ is the input power, the $P_{mbx}$ is the multimode bending output power of the TE${}_{x}$ (*x* is the mode order of the signal) mode, and the $L_{mbx}$ represents the bending loss coefficient of the TE${}_{x}$ mode via the multimode bending. In addition, inter-mode crosstalk noise occurs when TE${}_{0}$, TE${}_{1}$, …, and TE${}_{x}$ modes pass through a multimode bending. When the input is TE${}_{m}$ mode, the output is TE${}_{m}$ mode and a small amount of TE${}_{n}$ mode. Equation (2) describes corresponding inter-mode crosstalk noise, where ${Cmb}_{mn}$ represents the inter-mode crosstalk noise coefficient of TE${}_{m}$ mode converted to TE${}_{n}$ mode.

(1) $$P_{mbx}=P_{in}\cdot L_{mbx}$$

(2) $$P_{mb,mn}=P_{in}\cdot {Cmb}_{mn}$$

**Figure 3:**
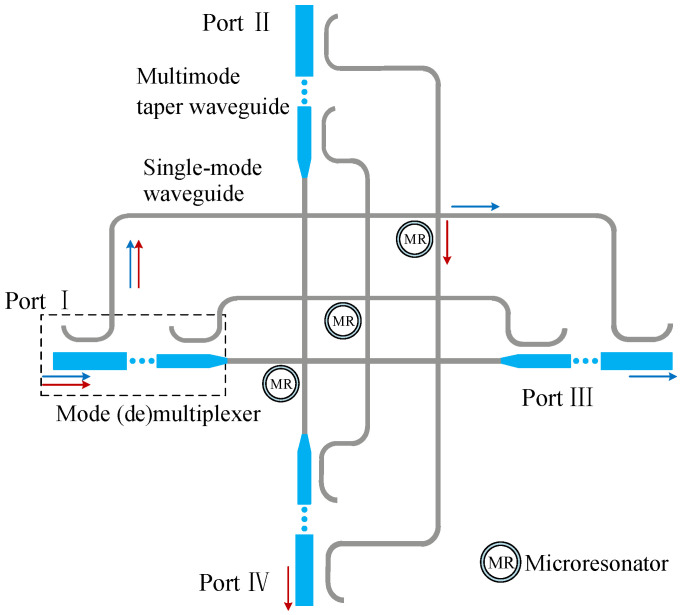
Multimode switching element. The red and blue arrows represent the trajectories of TE${}_{x}$ mode transmission in different states of MR.

In the traditional single-mode optical router, the microring resonator acts as a crucial optical element for transmitting optical signals, which can change or maintain the transmission direction of optical signals. At present, most of the microring resonators are composed of single-mode waveguides. In order to achieve multimode transmission, it is necessary to design an optical device that can maintain or change the transmission direction of multiple modes. The symmetrical switching element proposed in this paper can realize this function, and its schematic diagram is shown in Figure 3.

It can be seen from Figure 3 that the multimode switching element includes mode (de)mu-ltiplexers, single-mode waveguides, microring resonators, single-mode bendings, and single-mode waveguide crossings. When the signals carry multiple modes are input from Port I, they are first directly demultiplexed to the fundamental modes through the mode demultiplexer. Immediately after, their transmission directions are changed by the microring resonators, which determine whether the fundamental modes are transferred to Port III or Port IV. Microring resonators have ON state and OFF state. When the microring resonator is in the ON state, it means that the wavelength of the input optical signal is equal to the resonant wavelength of the microring resonator, and then the optical signal is coupled into the microring and transmitted to Port IV. In another case, when the microring resonator is in the OFF state, it indicates that the wavelength of the input optical signal is not equal to the resonant wavelength of the microring resonator. Instead of being coupled into the microring, the optical signal is transmitted directly to Port III.

Specifically, the red and blue arrows shown in Figure 3 represent the trajectory diagram of the TE${}_{x}$ mode transmission. Firstly, the TE${}_{x}$ mode is input from Port I, which is demultiplexed to the fundamental mode by the mode demultiplexer. Subsequently, the fundamental mode pass through the single-mode bending and the microring resonator. When the microring resonator is in the OFF state, the fundamental mode is transmitted directly to Port III through the microring resonator for mode multiplexing, which is restored to the TE${}_{x}$ mode. Finally, the TE${}_{x}$ mode is output from Port III and transmitted to the next multimode optical device, as shown by the blue arrow in Figure 3. When the microring resonator is in the ON state, the fundamental mode signal is coupled into the microring and transmitted to Port IV. After that, the fundamental mode is restored to the TE${}_{x}$ mode, which is output by Port IV of the multimode switching element and transmitted to the next multimode optical device. This process is illustrated by the red arrow in Figure 3.

Thus, by controlling the state of the microring resonator (ON state, OFF state), the multimode switching element can control the high-order mode output from Port III or Port IV. Therefore, the multimode switching element can overcome the disadvantage that the traditional single-mode CSE can only transmit the fundamental mode. It can be used to transmit different high-order modes and control their transmission direction.

The insertion loss and crosstalk noise are generated when the TE${}_{x}$ mode passes through the multimode switching element. However, transmission loss varies with input port or output port. The source of insertion loss and crosstalk noise can be analyzed from the following aspects:

(1) The insertion loss and crosstalk noise generated by mode-division (de)multiplexing. We can see this from the structure of multimode switching element in Figure 3, where the mode (de)multiplexer is designed and implemented based on the asymmetric directional coupler (ADC), and each mode (de)multiplexer contains *N*-1 ADCs, where *N* is the number of modes supported by the multimode switching element.

**Figure 4:**
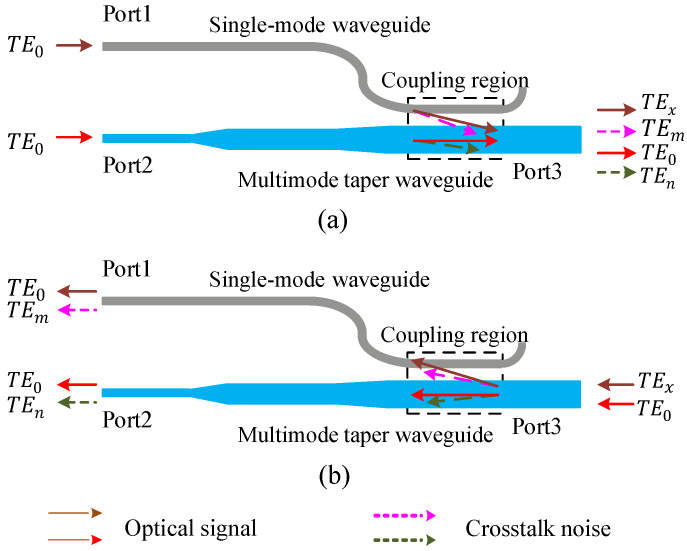
Mode (de)multiplexer for TE${}_{x}$ mode. (**a**) The process of mode multiplexer. (**b**) The process of mode demultiplexer.

Figure 4 shows a mode (de)multiplexer for TE${}_{x}$ mode, which consists of a single-mode waveguide and a multimode taper waveguide. As shown in the brown and red arrows in Figure 4a, when the function of mode multiplexing is realized, the fundamental mode signal with the same wavelength are input from Port 1 and Port 2, respectively. When the TE${}_{0}$ mode is input from Port 1, if the phase matching condition is satisfied, the TE${}_{0}$ mode is converted into different high-order modes through the coupling region due to different widths of the multimode taper waveguide; hereafter, the TE${}_{x}$ mode output from Port 3. The output power of the TE${}_{x}$ mode from Port 3 can be given by Equation (3), where the $L_{mxx}$ is the mode multiplexed loss coefficient of the TE${}_{0}$ mode converted to the TE${}_{x}$ mode. When the TE${}_{0}$ mode is input from Port 2, it is output directly from Port 3 without mode conversion, and the corresponding output power can be calculated by Equation (4). The $L_{mx0}$ is the loss coefficient of the TE${}_{0}$ mode passing through the multimode taper waveguide during mode multiplexing. Utilizing the symmetrical characteristics of directional coupler, the inverse process of the multiplexing process can realize the mode demultiplexing function. As shown by the red and brown arrows in Figure 4b, TE${}_{0}$ mode and TE${}_{x}$ mode input from Port 3 are demultiplexed. At last, TE${}_{0}$ mode will be obtained at Port 1 and Port 2, and their corresponding output power can be given by Equations (5) and (6), respectively. The $L_{dmxx}$ is the demultiplexing loss coefficient of the TE${}_{x}$ mode restored to the TE${}_{0}$ mode, and $L_{dmx0}$ is the loss coefficient of the TE${}_{0}$ mode passing through multimode taper waveguide during mode demultiplexing.

(3) $$P_{mxx}=P_{in}\cdot L_{mxx}$$

(4) $$P_{mx0}=P_{in}\cdot L_{mx0}$$

(5) $$P_{dmxx}=P_{in}\cdot L_{dmxx}$$

(6) $$P_{dmx0}=P_{in}\cdot L_{dmx0}$$

In addition, mode crosstalk noise inevitably occurs in mode-division (de)multiplexing. For example, when the TE${}_{0}$ mode is input from Port 1 and converted to target TE${}_{x}$ mode through the coupling region, a small part of TE${}_{0}$ mode is converted to the undesired TE${}_{j}$ mode, and the corresponding crosstalk noise can be illuminated as Equation (7). $C_{0jmx}$ is the crosstalk noise coefficient of mode-division multiplexing. Correspondingly, crosstalk noise will also occur when the mode is demultiplexed, that is, when the TE${}_{n}$ mode is restored to TE${}_{0}$ mode, part of the TE${}_{n}$ mode is converted to TE${}_{i}$ mode. The mode crosstalk noise can be illuminated as Equation (8), where $C_{nidmx}$ is the mode crosstalk noise coefficient of mode-division demultiplexing.

(7) $$P_{cmx}=P_{in}\cdot C_{0jmx}\;j\in \left\{1,2,3,\ldots \right\}$$

(8) $$P_{cdmx}=P_{in}\cdot C_{nidmx}\;n\neq i,\;n\in \left\{0,1,2,\ldots \right\},\;i\in \left\{1,2,3,\ldots \right\}$$

**Figure 5:**
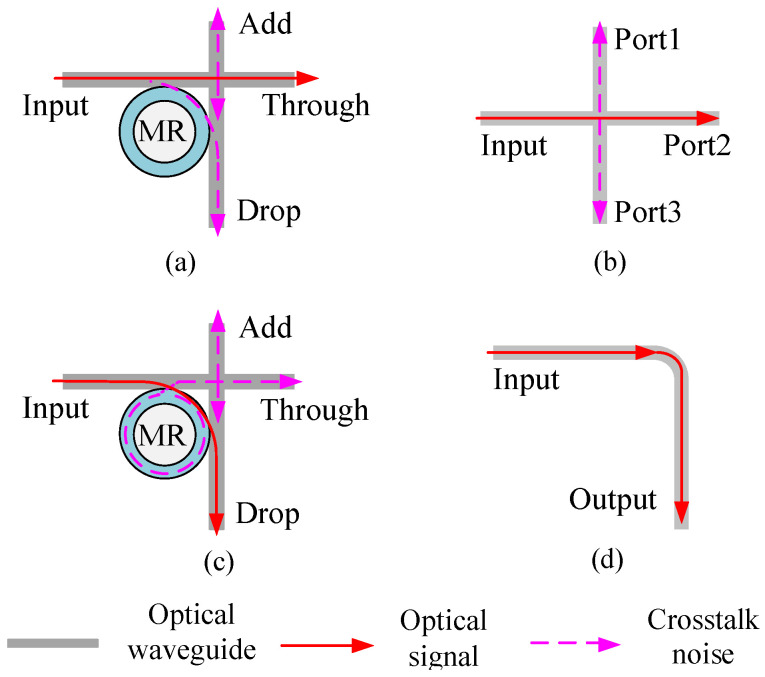
(**a**) Single-mode CSE in the OFF state. (**b**) Single-mode waveguide crossing. (**c**) Single-mode CSE in the ON state. (**d**) Single-mode bending.

(2) The insertion loss and crosstalk noise generated by the basic optical switching elements (BOSEs), the single-mode waveguide crossings, and the single-mode bendings. As shown in Figure 5, they are the BOSEs, single-mode bending and single-mode waveguide crossing of the single-mode optical router. The BOSEs used in this paper is the basic 1×2 crossing switching element (CSE), as shown in Figure 5a,c.

As for the 1×2 CSE, it is composed of two single-mode waveguides and a microring resonator. When the microring resonator is in the OFF state, the fundamental mode is transmitted directly to the Through port, as shown in Figure 5a. Therefore, the output power of the Through port can be calculated by Equation (9). On the contrary, when the microring resonator is in the ON state, the fundamental mode is coupled into the microring and transmitted to the Drop port, as shown in Figure 5c; the output optical power from the Drop port can be calculated by Equation (10). $L_{sp1}$ is the power loss of each parallel switching element (PSE) in the OFF state, and $L_{sc2}$ is the power loss of each CSE in the ON state. For the 1×2 CSE, when the microring resonator is in the ON state, the output power of the Through port and the Add port can be calculated by Equations (11) and (12), respectively. $K_{c}$ is the crosstalk noise coefficient of the waveguide crossing. When the microring resonator is in the OFF state, the output power of the Drop port and the Add port can be calculated by Equations (13) and (14), respectively. $C_{p,on}$ is the crosstalk noise coefficient of each 1×2 PSE in the ON state, and $C_{p,off}$ is the crosstalk noise coefficient of each PSE in the OFF state [30].

(9) $$P_{T,c,off}=P_{in}\cdot L_{sp1}L_{sc}$$

(10) $$P_{D,c,on}=P_{in}\cdot L_{sc2}$$

(11) $$P_{T,c,on}=P_{in}\cdot C_{p,on}L_{sc}$$

(12) $$P_{A,c,on}=P_{in}\cdot C_{p,on}K_{c}$$

(13) $$P_{D,c,off}=P_{in}\cdot \left(C_{p,off}+L_{sp1}^{2}K_{c}\right)$$

(14) $$P_{A,c,off}=P_{in}\cdot L_{sp1}K_{c}$$

Figure 5b shows the single-mode waveguide crossing. When the fundamental mode enters the single-mode waveguide crossing from the Input port, it outputs directly from Port 2, and the power output can be established as Equation (15). $L_{sc}$ is the single-mode waveguide crossing loss coefficient. Figure 5d is the single-mode bending; when the fundamental mode is input from the Input port, the power output from the Output port can be established as Equation (16), where $L_{sb}$ describes single-mode bending loss coefficient. Crosstalk noise will be produced when two optical signals pass through a waveguide crossing at the same time. When the optical signal enters the waveguide crossing from the Input port, the output power of Port 1 and Port 3 can be described as Equation (17).

(15) $$P_{sc}=P_{in}\cdot L_{sc}$$

(16) $$P_{sb}=P_{in}\cdot L_{sb}$$

(17) $$P_{port1}=P_{port3}=P_{in}\cdot K_{c}$$

Through the above analysis of insertion loss and crosstalk noise sources, we can obtain the insertion loss and crosstalk noise between port pairs of the multimode switching element. Since the optical devices are used to transmit multiple modes, the insertion loss will vary with the input mode. When the TE${}_{k}$ mode is input from Port I of this multimode switching element and finally restored to the TE${}_{k}$ mode output from the Port III, the insertion loss generated by this process can be obtained by Equation (18), $k\in \left\{1,2,\ldots ,N-1\right\}$.

As can be seen from Figure 3, when the input port is Port III, and the corresponding output port is Port I, the insertion loss can also be calculated by Equation (18). In addition, due to the symmetry of the device, when the input port is Port II, the corresponding output port is Port IV, and when the input port is Port IV, the corresponding output port is Port II; their insertion losses are equal to Equation (18). Similarly, when the TE${}_{k}$ mode is input from Port III of this multimode switching element, finally restored to the TE${}_{k}$ mode and output from Port IV, the insertion loss generated by this process can be established as Equation (20). For TE${}_{0}$ mode, when it is input from Port I, Port III, Port II, and Port IV of the multimode switching element, the corresponding output port is Port III, Port I, Port IV, and Port II, respectively; the insertion loss can be calculated using Equation (19). Moreover, when the TE${}_{0}$ mode is input from the Port I and the corresponding output from the Port IV, the corresponding insertion loss can be obtained by Equation (21).

(18) $$P_{IIIk}=P_{in}\cdot L_{dmxk}L_{sc}^{N}L_{sp1}L_{sb}^{2}L_{mxk}$$

(19) $$P_{III0}=P_{in}\cdot L_{dmx0}L_{sc}^{N}L_{sp1}L_{mx0}$$

(20) $$P_{IVk}=P_{in}\cdot L_{dmxk}L_{sc}^{2k}L_{sc2}L_{sb}^{2}L_{mxk}$$

(21) $$P_{IV0}=P_{in}\cdot L_{dmx0}L_{sc2}L_{mx0}$$

**Figure 6:**
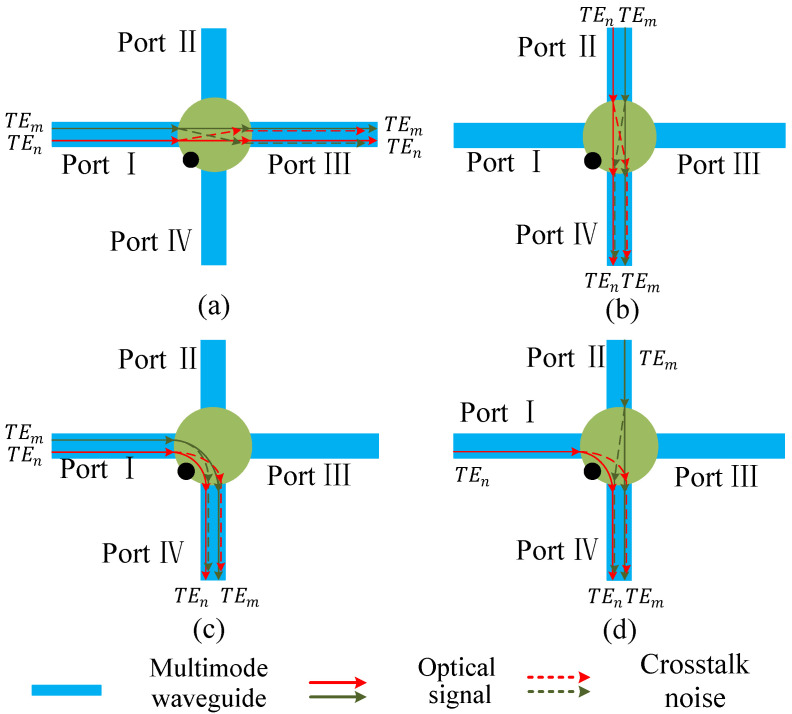
The four communication situations where two modes are output from the same port of the multimode switching element. (**a**–**c**) show situations that the two modes are input from the same port. (**d**) shows situation that the two modes are input from different ports.

**Figure 7:**
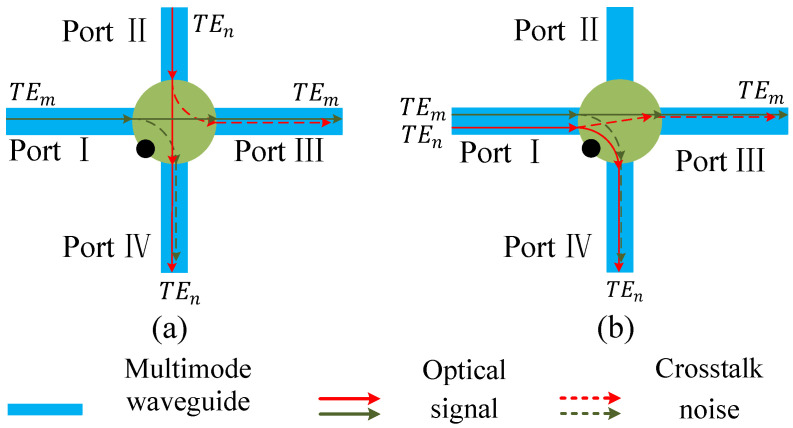
The two communication situations where the two modes are output from different ports of the multimode switching element. (**a**) The two modes are input from different ports. (**b**) The two modes are input from same ports.

Compared with the insertion loss, the crosstalk noise analysis of multimode switching element is more complicated, and it is mainly divided into 6 situations shown in Figure 6 and Figure 7. Figure 6 shows the four communication situations where two modes are output from the same port of the multimode switching element. Figure 7 describes the two communication situations where two modes are output from different ports of the multimode switching element. Among them, Figure 6a–c show the situations that the two modes enter from the same port of the multimode switching element and then output from the same port. Figure 6d displays that the two modes are input from different ports of the multimode switching element, but output from the same port. Since the single-mode optical waveguide has a suppressive effect on higher-order modes, this paper ignores the crosstalk noise generated by the mode demultiplexing process.

Combined with above analysis on the source of crosstalk noise, in the three cases of Figure 6a–c, after the two modes are restored to the TE${}_{0}$ mode, they have no intersection in the single-mode waveguide. In other words, the crosstalk noise between them only comes from the mode multiplexing process, and the corresponding crosstalk noise $C_{1,1}$, $C_{1,2}$, and $C_{1,3}$ can be illuminated as Equations (22) and (23). When the two modes are input from different ports of the multimode switching element and output from the same port, the crosstalk noise $C_{2,1}$ generated by the TE${}_{m}$ mode to the TE${}_{n}$ mode can be illuminated as Equation (24). Correspondingly, the crosstalk noise $C_{2,2}$ generated by TE${}_{n}$ mode to TE${}_{m}$ mode can be obtained by Equation (25).

(22) $$\left[\begin{array}{c} C_{1,1}\\ C_{1,2}\\ C_{1,3} \end{array}\right]=P_{in}\cdot \left[\begin{array}{cc} L_{dmx0}L_{sc}^{N}C_{0nmx} & 0\\ L_{dmx0}L_{sc}^{N}C_{0nmx} & 0\\ 0 & L_{dmx0}C_{0nmx} \end{array}\right]\cdot \left[\begin{array}{c} L_{sp1}\\ L_{sc2} \end{array}\right]\;m=0$$

(23) $$\left[\begin{array}{c} C_{1,1}\\ C_{1,2}\\ C_{1,3} \end{array}\right]=P_{in}\cdot \left[\begin{array}{cc} L_{dmxm}L_{sc}^{N}L_{sb}^{2}C_{mnmx} & 0\\ L_{dmxm}L_{sc}^{N}L_{sb}^{2}C_{mnmx} & 0\\ 0 & L_{dmxm}L_{sc}^{2m}L_{sb}^{2}C_{mnmx} \end{array}\right]\cdot \left[\begin{array}{c} L_{sp1}\\ L_{sc2} \end{array}\right]\;m\neq 0$$

(24) $$\begin{array}{c} \left[\begin{array}{c} C_{2,1}^{a}\\ C_{2,1}^{b}\\ C_{2,1}^{c}\\ C_{2,1}^{d} \end{array}\right]=P_{in}\cdot \left[\begin{array}{cc} L_{dmxm}L_{sc}^{N}L_{sb}^{2}C_{m0mx} & 0\\ L_{dmxm}L_{sc}^{N}C_{0nmx} & L_{dmxm}L_{sb}L_{sc}^{N+n-2}K_{c}L_{mxn}\\ L_{dmxm}L_{sc}^{N}L_{sb}^{2}C_{mnmx} & L_{dmxm}L_{sc}^{N+n-m-2}L_{sb}^{2}K_{c}L_{mxn}\\ L_{dmxm}L_{sc}^{N}L_{sb}^{2}C_{mnmx} & 0 \end{array}\right]\cdot \left[\begin{array}{c} L_{sp1}\\ L_{sc2} \end{array}\right]\;\\ a:n=0;b:m=0,n>m;c:n>m,m\neq 0;d:n<m \end{array}$$

(25) $$\begin{array}{c} \left[\begin{array}{c} C_{2,2}^{a}\\ C_{2,2}^{b}\\ C_{2,2}^{c}\\ C_{2,2}^{d} \end{array}\right]=P_{in}\cdot \left[\begin{array}{cc} 0 & L_{dmx0}C_{0mmx}\\ 0 & L_{dmxn}L_{sc}^{2n}L_{sb}^{2}C_{nmmx}\\ L_{dmxn}L_{sc}^{n}L_{sb}K_{c}L_{mx0} & L_{dmxn}L_{sc}^{2n}L_{sb}^{2}C_{n0mx}\\ L_{dmxn}L_{sc}^{m+n}L_{sb}^{2}K_{c}L_{mxm} & L_{dmxn}L_{sc}^{2n}L_{sb}^{2}C_{nmmx} \end{array}\right]\cdot \left[\begin{array}{c} L_{sp1}\\ L_{sc2} \end{array}\right]\;\\ a:n=0;b:n\neq 0,m>n;c:n\neq 0,m=0;d:m\neq 0,n>m \end{array}$$

Figure 7a,b describe the situations where the two modes are output from different ports of the multimode switching element. In Figure 7a, the two modes will inevitably intersect in the single-mode waveguide inside the multimode switching element, the corresponding crosstalk noise $C_{3,1}$ and $C_{3,2}$ generated by the TE${}_{m}$ mode to the TE${}_{n}$ mode can be expressed as Equation (26) and (27). Equation (26) represents the crosstalk noise generated from the TE${}_{m}$ mode to the TE${}_{n}$ mode, and Equation (27) is the crosstalk noise generated from the TE${}_{n}$ mode to the TE${}_{m}$ mode.

(26) $$\begin{array}{c} \left[\begin{array}{c} C_{3,1}^{a}\\ C_{3,1}^{b}\\ C_{3,1}^{c}\\ C_{3,1}^{d}\\ C_{3,1}^{e} \end{array}\right]=P_{in}\cdot \left[\begin{array}{cc} L_{dmx0}\left(C_{pse,off}+L_{sp1}^{2}K_{c}\right) & 0\\ L_{dmxm}L_{sc}^{m}L_{sp1}L_{sb}K_{c} & 0\\ 0 & L_{dmx0}L_{sc}^{n}L_{sp1}L_{sb}K_{c}\\ 0 & L_{dmxm}L_{sc}^{m+n}L_{sp1}L_{sb}^{2}K_{c}\\ 0 & L_{dmxm}L_{sc}^{2m}L_{sb}^{2}\left(C_{pse,off}+L_{sp1}^{2}K_{c}\right) \end{array}\right]\\ \cdot \left[\begin{array}{c} L_{mx0}\\ L_{mxn} \end{array}\right]\;a:m=0,n=0;b:m\neq 0,n=0;c:m=0,n\neq 0;\\ d:m\neq 0,n\neq 0,m\neq n;e:m\neq 0,n\neq 0,m=n \end{array}$$

(27) $$\begin{array}{c} \left[\begin{array}{c} C_{3,2}^{a}\\ C_{3,2}^{b}\\ C_{3,2}^{c}\\ C_{3,2}^{d}\\ C_{3,2}^{e} \end{array}\right]=\left[\begin{array}{cc} L_{dmx0}L_{sc}^{2(N-1)}\left(K_{c}+L_{sc}^{2}C_{pse,off}\right) & 0\\ 0 & L_{sc}^{2(N-1)-m}L_{sp1}L_{sb}K_{c}\\ 0 & L_{dmxn}L_{sc}^{2(N-1)-n}L_{sp1}L_{sb}K_{c}\\ 0 & L_{dmxn}L_{sc}^{2(N-1)-n-m}L_{sp1}L_{sb}^{2}K_{c}\\ 0 & L_{dmxn}L_{sc}^{2(N-1-m)}L_{sb}^{2}\left(K_{c}+L_{sc}^{2}C_{pse,off}\right) \end{array}\right]\\ \cdot \left[\begin{array}{c} L_{mx0}\\ L_{mxm} \end{array}\right]\cdot P_{in}\;a:m=0,n=0;b:m\neq 0,n=0;c:m=0,n\neq 0;d:m\neq 0,\\ n\neq 0,m\neq n;e:m\neq 0,n\neq 0,m=n \end{array}$$

For the situation shown in Figure 7b, the two modes are input from the same port of the multimode switching element, but they are output from different ports. When n>m, the two modes will produce crosstalk noise in the single-mode waveguide part of the mode switching element, and the corresponding crosstalk noise $C_{4,1}$ and $C_{4,2}$ can be illuminated as Equations (28) and (29). Equation (28) indicates the crosstalk noise generated from the TE${}_{m}$ mode to the TE${}_{n}$ mode, and Equation (29) indicates the crosstalk noise generated from the TE${}_{n}$ mode to the TE${}_{m}$ mode.

(28) $$C_{4,1}=\left\{\begin{array}{cc} P_{in}L_{dmx0}L_{sc}^{n}L_{sp1}L_{sb}K_{c}L_{mxn} & m=0\\ 0 & n<m\\ P_{in}L_{dmxm}L_{sc}^{m+n}L_{sp1}L_{sb}^{2}K_{c}L_{mxn} & m\neq 0,n>m \end{array}\right.$$

(29) $$C_{4,2}=\left\{\begin{array}{cc} P_{in}L_{dmxn}L_{sc}^{N+n-2}L_{sc2}L_{sb}K_{c}L_{mx0} & m=0\\ 0 & n<m\\ P_{in}L_{dmxn}L_{sc}^{N+n-m-2}L_{sc2}L_{sb}^{2}K_{c}L_{mxm} & m\neq 0,n>m \end{array}\right.$$

When a traditional single-mode optical router is working, there may be multiple optical signals inside. However, some specific microring resonators is only in the ON state on the link from a certain port to a certain port and in the OFF state on the other port-to-port link. Therefore, by adjusting the position of the multimode switching element, the design of the MDM-OR that supports MDM can be realized.

**Figure 8:**
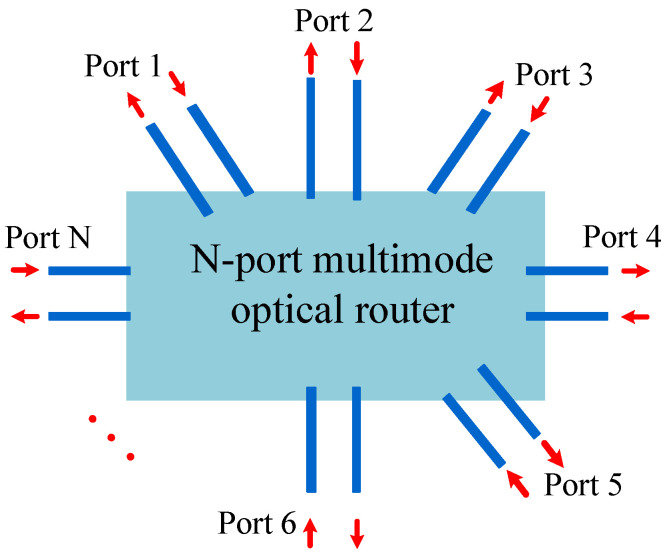
*N*-port MDM-OR.

Figure 8 shows a simplified model of an *N*-port optical router. The optical router has *N* bidirectional ports, namely Port 1, Port 2, …, Port *N*. When the optical signal is transmitted from Port *i* to Port *j* of the MDM-OR, the optical signal power $P_{i,j}$ can be obtained by Equation (30). $P_{in,i}$ is the power, which injects into Port *i* of the MDM-OR. $L_{i,j(x,y)}$ is defined as the transmission loss coefficient from Port *i* to the Port *j* of the *N*-port MDM-OR. The process produces total crosstalk noise $C_{i,j}\left(x,y\right)$, which can be calculated by Equation (31), where $P_{k}\left(x,y\right)$ is defined as the signal power, which is injected into the MDM-OR from Port *k*, and $K_{i,j,k}\left(x,y\right)$ indicates the crosstalk noise coefficient of the optical signal introduced by $P_{k}\left(x,y\right)$ into the MDM-OR.

(30) $$P_{i,j}=P_{in,i}\cdot L_{i,j(x,y)}\;i,j\in \left\{0,1,2,\ldots ,N-1\right\}$$

(31) $$C_{i,j}\left(x,y\right)=\sum_{k=0}^{N-1}P_{k}\left(x,y\right)\cdot K_{i,j,k}\left(x,y\right)\;i,j,k\in \left\{0,1,2,\ldots ,N-1\right\}$$

OSNR represents the ratio of signal power to noise power. More importantly, it is a crucial parameter to measure the performance of an optical router. Equation (32) can be used to calculate OSNR. Based on Equation (32), we can establish the BER, which is presented in Equation (33). BER is the ratio of received error messages to the total amount of information transmitted, which is an important index to judge the accuracy of information transmission.

(32) $$OSNR=10\log_{10}\left(\frac{P_{\left(x_{0},y_{0}\right),\left(x_{1},y_{1}\right)}}{P_{N\left(x_{0},y_{0}\right),\left(x_{1},y_{1}\right)}}\right)$$

(33) $$BER=\frac{1}{2}erfc\left(\frac{1}{2}\sqrt{OSNR}\right)$$

## 3. Results

**Figure 9:**
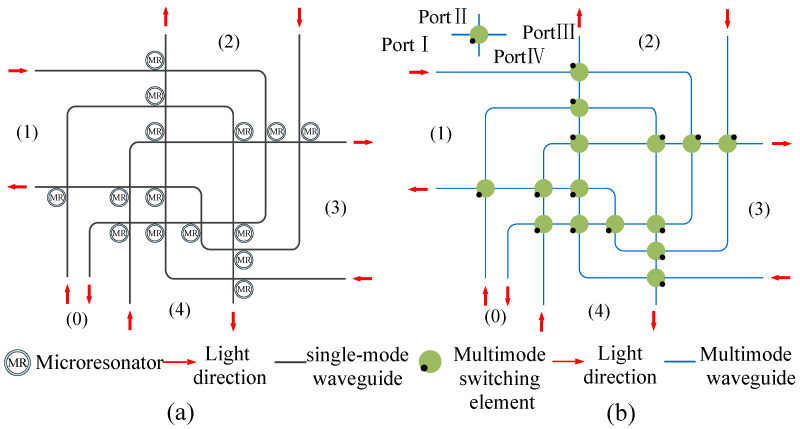
(**a**) The five-port single-mode optical router. (**b**) The five-port MDM-OR that supports MDM.

In this section, based on the replacement scheme of Figure 1, we use multimode waveguide to replace the traditional single-mode waveguide, multimode bending to replace the single-mode bending, multimode waveguide crossing to replace the single-mode waveguide crossing, and multimode switching element to replace the 1×2 CSE. Finally, the five-port MDM-OR, which supports MDM, is shown in Figure 9b. The optical router shown in Figure 9a is a five-port optical router, which is the single-mode optical router before replacement. In addition, the five-port MDM-OR is a general 5×5 all-pass optical router, that is, the mode can be transferred from one port to any other port. Different from a traditional single-mode optical router, MDM-OR can not only transmit TE${}_{0}$ mode but also transmit other modes at the same time. The five-port MDM-OR is just a concrete implementation of our method. Based on this replacement scheme, we can build *N*-port MDM-ORs.

We utilize the calculation models of insertion loss, crosstalk noise, OSNR, and BER in the previous section and use MATLAB to obtain the transmission loss, crosstalk noise, OSNR, and BER between different port pairs of this five-port MDM-OR in the input 1550 nm TE${}_{0}$, TE${}_{1}$, and TE${}_{2}$ mode. Additionally, in the simulation example, the input power is 0 dBm and other parameters used are shown in Table 1 and Table 2.

**Figure 10:**
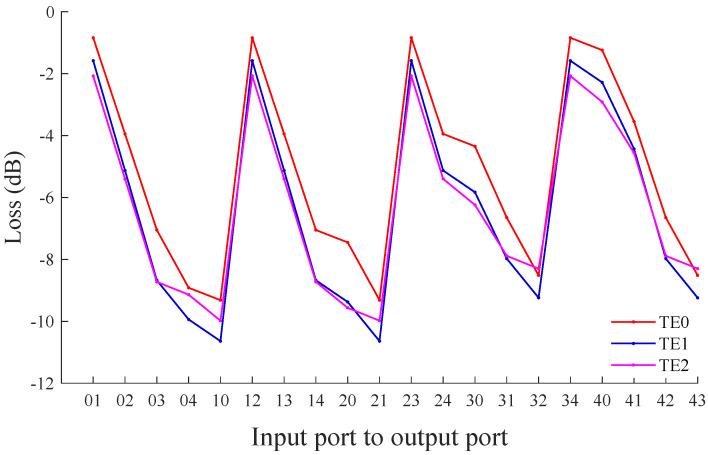
The transmission loss between different port pairs of the multimode router based on the TE${}_{0}$, TE${}_{1}$ and TE${}_{2}$ mode.

According to the insertion loss models of different multimode optical devices in the previous section, the transmission loss between different port pairs of the five-port MDM-OR is calculated. Figure 10 shows the transmission loss between different port pairs of this router when the TE${}_{0}$, TE${}_{1}$, and TE${}_{2}$ mode is input, where 12 in the abscissa represents the TE${}_{0}$ mode being transmitted from Port 1 to Port 2. It can be seen from Figure 10 that when the optical router transmits the TE${}_{0}$ mode, the transmission loss between the different port pairs of the MDM-OR is in the range of (−9.31, −0.84) dB. According to the same method, we can obtain the transmission loss between different port pairs when this router inputs TE${}_{1}$ mode. We can find that the maximum transmission loss between different port pairs is −10.64 dB, and the minimum is −1.58 dB. When the TE${}_{2}$ mode is input, its transmission loss range is from −9.98 dB to −2.07 dB.

From Figure 10, we can see that the transmission loss of the corresponding port pairs are not much different when the five-port MDM-OR transmits different mode signals. It can be seen from Table 1 that the higher the order mode signals that pass through the multimode waveguide crossing and the multimode bending, the higher the insertion loss will be. However, the multimode switching element we designed can adjust this situation so that the transmission loss between different port pairs in different modes is at an average level.

**Figure 11:**
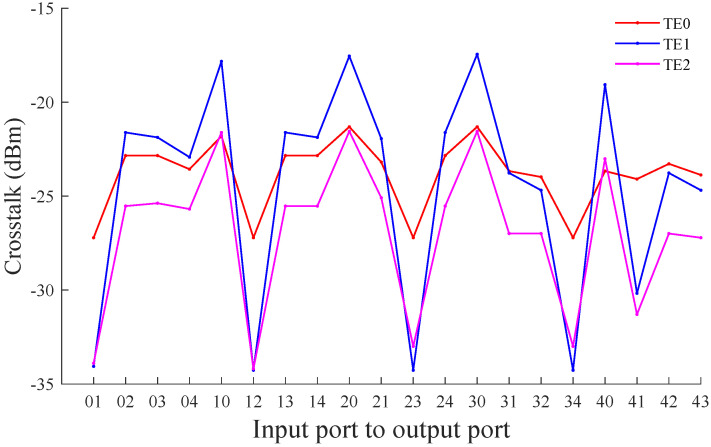
The crosstalk noise power between different port pairs of the multimode router based on the TE${}_{0}$, TE${}_{1}$ and TE${}_{2}$ mode.

By using the crosstalk noise calculation model proposed in the previous section, we can obtain the crosstalk noise power accumulated on the main signal when different modes are transmitted between different port pairs in the MDM-OR. The specific simulation results are shown in Figure 11. Figure 11 shows the crosstalk noise power accumulated on the TE${}_{0}$ mode during transmission between different port pairs in the MDM-OR. It can be seen from Figure 11 that when the TE${}_{0}$ mode is transmitted between different port pairs, the crosstalk noise power accumulated on it is different, and the crosstalk noise power is in the range of (−27.21, −21.31) dBm. When the TE${}_{1}$ mode is transmitted between different port pairs in the MDM-OR, the crosstalk noise power accumulated on it ranges from −34.27 dBm to −17.45 dBm. Correspondingly, when the TE${}_{2}$ mode is transmitted between different port pairs in the MDM-OR, the range of crosstalk noise power is −34.19 dBm to −21.55 dBm.

**Figure 12:**
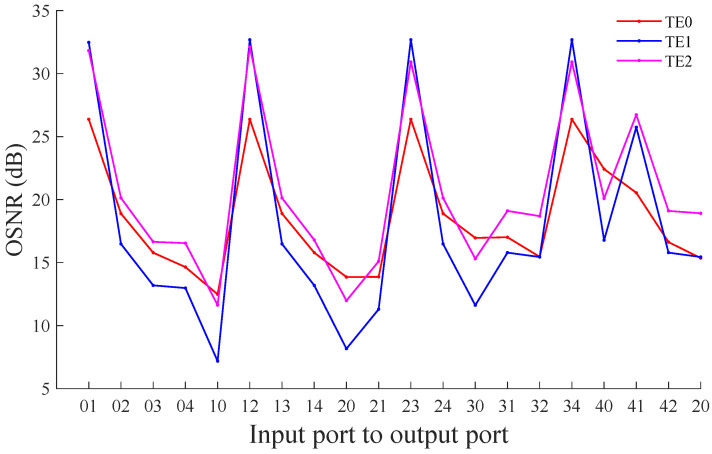
The OSNR between different port pairs of the multimode router based on the TE${}_{0}$, TE${}_{1}$, and TE${}_{2}$ mode.

After obtaining the transmission loss and the crosstalk noise power of different port pairs of the MDM-OR under different modes, the OSNR of different modes can be calculated using Equation (33). In this paper, OSNR is calculated for continuous wave transmission. Figure 12 shows the OSNR when the TE${}_{0}$, TE${}_{1}$, and TE${}_{2}$ mode passes through different port pairs of the MDM-OR. From the figure, it can be found that the OSNR transmitted by TE${}_{0}$ mode between different port pairs of the MDM-OR is different. In general, the OSNR of the TE${}_{0}$ mode is excellent, the maximum OSNR can reach 26.37 dB, and the minimum OSNR can also reach 12.49 dB. For TE${}_{1}$ mode, the OSNR of transmission in this case ranges from 7.19 dB to 32.68 dB. In the five-port MDM-OR, the OSNR of TE${}_{2}$ mode is also different, among which the maximum value is 32.11 dB and the minimum value is 11.63 dB.

**Figure 13:**
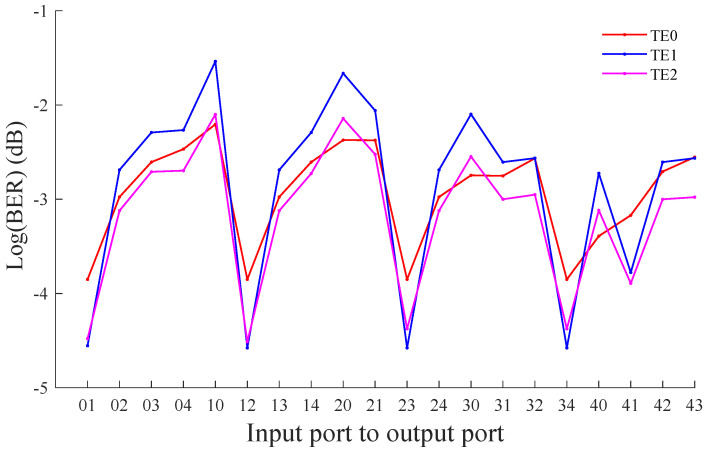
The BER between different port pairs of the multimode router based on the TE${}_{0}$, TE${}_{1}$, and TE${}_{2}$ mode.

According to the obtained OSNR, Equation (33) is used to calculate the BER in different modes. Figure 13 describes the BER of TE${}_{0}$, TE${}_{1}$, and TE${}_{2}$ modes through different port pairs of MDM-OR. It can be found that the BER for the TE${}_{0}$, TE${}_{1}$, and TE${}_{2}$ mode ranges from −3.85 dB to −2.10 dB, −4.58 dB to −1.54 dB, and −4.51 dB to −2.10 dB, respectively. In this paper, BER is calculated according to 10 Gb/s bit rate, and the modulation mode is non-return-to-zero (NRZ).

It is believed that, with research into multimode bending and multimode waveguide crossing and the continuous development of fabrication technology, the insertion loss and crosstalk noise of different high-order modes passing through them will be reduced. Moreover, with the extensive research into mode (de)multiplexer, the corresponding insertion loss and crosstalk noise of mode (de)multiplexing will be further reduced. Therefore, the transmission loss and crosstalk noise of MDM-OR designed by general model will be greatly reduced, and its performance will be greatly improved.

## 4. Conclusions

Since multimode waveguides can transmit different modes, MDM technology is considered an effective method to further increase the capacity of the communication systems. The optical router supporting MDM is an important part of the ONoC implements MDM. With the rapid development of MDM, OR based on MDM came into being. In this paper, we propose a model of MDM-OR that can realize MDM. After that, using this model, we construct the MDM-OR corresponding to the single-mode five-port optical router, and the transmission loss, crosstalk noise power, OSNR, and BER of the port pairs are analyzed. The simulation results show that when TE${}_{0}$, TE${}_{1}$, and TE${}_{2}$ modes of 1550 nm are input, the transmission loss of the port pairs corresponding to the five-port MDM-OR is less than −10.64 dB. In this case, the crosstalk noise power of the port pairs corresponding to the five-port MDM-OR is less than −17.45 dBm. Correspondingly, in the five-port MDM-OR, the OSNR of TE${}_{0}$, TE${}_{1}$, and TE${}_{2}$ modes can reach up to 32.68 dB after passing through different channels, the minimum value of BER is −4.58 dB. From the simulation results, we can see that the MDM-OR based on the model in this paper has small transmission loss, and there will be a great prospect in the future in the field of ONoC that supports MDM.

## Figures and Tables

**Table 1:** Loss cofficients.

Parameter	Value	Reference
$L_{sb}$	−0.005 dB/90${}^{\circ }$	[31]
$L_{sc}$	−0.04 dB	[32]
$L_{sc2}$	−0.5 dB	[33]
$L_{sp1}$	−0.005 dB	[33]
$L_{mb0}$	−0.4 dB	[21]
$L_{mb1}$	−0.7 dB	[21]
$L_{mb2}$	−0.84 dB	[21]
$L_{dmx0}$	−0.17 dB	[34]
$L_{dmx1}$	−0.27 dB	[34]
$L_{dmx2}$	−0.13 dB	[34]
$L_{mx0}$	−0.17 dB	[34]
$L_{mx1}$	−0.13 dB	[34]
$L_{mx2}$	−0.09 dB	[34]

**Table 2:** Crosstalk cofficients.

Parameter	Value	Reference
$K_{c}$	−40 dB	[31]
$C_{pse,off}$	−20 dB	[32]
$C_{pse,on}$	−25 dB	[33]
$C_{01mx}$	−35 dB	[34]
$C_{02mx}$	−48.8 dB	[34]
$C_{10mx}$	−43 dB	[34]
$C_{12mx}$	−37.3 dB	[34]
$C_{20mx}$	−25.4 dB	[34]
$C_{21mx}$	−39.9 dB	[34]
${Cmb}_{01}$	−20 dB	[21]
${Cmb}_{02}$	−25 dB	[21]
${Cmb}_{10}$	−25 dB	[21]
${Cmb}_{12}$	−25 dB	[21]
${Cmb}_{20}$	−32 dB	[21]
${Cmb}_{21}$	−22 dB	[21]
